# Thioredoxin-Interacting Protein Regulates Glucose Metabolism and Affects Cytoplasmic Streaming in Mouse Oocytes

**DOI:** 10.1371/journal.pone.0070708

**Published:** 2013-08-19

**Authors:** Su-Yeon Lee, Hyun-Seo Lee, Eun-Young Kim, Jung-Jae Ko, Tae Ki Yoon, Woo-Sik Lee, Kyung-Ah Lee

**Affiliations:** 1 Department of Biomedical Science, College of Life Science, CHA University, Seoul, Korea; 2 DNA Repair Research Center, Chosun University, Gwangju, Korea; 3 Fertility Center, CHA Gangnam Medical Center, CHA University, Seoul, Korea; Institute of Zoology, Chinese Academy of Sciences, China

## Abstract

Thioredoxin-interacting protein (*Txnip*) regulates intracellular redox state and prompts oxidative stress by binding to and inhibiting Thioredoxin (*Trx*). In addition, via a *Trx*-independent mechanism, *Txnip* regulates glucose metabolism and thus maintains intracellular glucose levels. Previously, we found *Txnip* mRNA highly expressed in immature germinal vesicle (GV) oocytes, but currently there is no report describing the role of *Txnip* in oocytes. Therefore, we conducted the present study to determine the function of *Txnip* in mouse oocytes' maturation and meiosis by using RNA interference (RNAi) method. Upon specific depletion of *Txnip*, 79.5% of oocytes were arrested at metaphase I (MI) stage. Time-lapse video microscopy analysis revealed that the formation of granules in the oocyte cytoplasm increased concurrent with retarded cytoplasmic streaming after *Txnip* RNAi treatment. *Txnip* RNAi-treated oocytes had upregulated glucose uptake and lactate production. To confirm the supposition that mechanism responsible for these observed phenomena involves increased lactate in oocytes, we cultured oocytes in high lactate medium and observed the same increased granule formation and retarded cytoplasmic streaming as found by *Txnip* RNAi. The MI-arrested oocytes exhibited scattered microtubules and aggregated chromosomes indicating that actin networking was disturbed by *Txnip* RNAi. Therefore, we conclude that *Txnip* is a critical regulator of glucose metabolism in oocytes and is involved in maintaining cytoplasmic streaming in mouse oocytes.

## Introduction

In female mammals, oocytes undergo meiotic division and are arrested at meiotic prophase I, referred to as the germinal vesicle (GV) stage, in primordial follicles [1]. Under the influence of a luteinizing hormone surge, meiotic maturation restarts shortly before ovulation following the onset of puberty [2]. Meiotic maturation is comprised of meiosis I and meiosis II [3]. In meiosis I, oocytes undergo GV breakdown (GVBD) and progress to metaphase I (MI) [4]. Without an intervening S phase, oocytes enter meiosis II and progress to metaphase II (MII) until the first polar body extrudes. Oocytes are arrested then again at the MII stage by cytostatic factor until fertilization [5].

In a previous study, we identified differentially expressed mRNAs between the GV and MII stages of mouse oocytes using annealing control primer-PCR [6]. Among differentially expressed genes, we found that Thioredoxin interacting protein *(Txnip)* mRNA highly expressed in GV oocytes compared to MII oocytes. *Txnip*, also known as Vitamin D3 upregulated protein 1 *(VDUP1)* and Thioredoxin binding protein 2 (*Tbp2*), was primarily identified as an inhibitor of Thioredoxin *(Trx)* [7]–[9] and TXNIP forms a mixed disulfide bond with TRX by disulfide exchange at catalytic active sites and inhibits its activity [10]. TRX is a critical protein conveying electrons from NADP(H) to protein disulfide bonds [11]. Thus, the interaction between TRX and TXNIP maintains the intracellular reducing environment [10].

In addition to TXNIP binding TRX, there are substantial evidences suggesting that *Txnip* has a major role in regulating glucose metabolism independent of *Trx* [12]–[14]. The *Txnip*-knockout mice exhibit problems in glucose and lipid metabolism such as low blood glucose, hyperlipidemia, and dysregulated response to fasting [15]–[18]. However, there is no report regarding the function of *Txnip* in the mouse oocytes. Therefore, the aims of the present study were the characterization of the expression of *Txnip* in mouse oocytes and the elucidation of functions of *Txnip* in oocytes.

## Materials and Methods

### Animals

All imprinting control region (ICR) mice were obtained from Koatech (Pyeoungtack, Korea) and maintained in the breeding facility at the CHA Stem Cell Institute of CHA University. All procedures described within this study were reviewed and approved by the Institutional Animal Care and Use Committee of CHA University and performed in accordance with the Guiding Principles for the Care and Use of Laboratory Animals.

### Collection of oocytes and follicular cells

For collection of GV oocytes from preovulatory follicles, 3-week-old female ICR mice were treated with 5 IU pregnant mare's serum gonadotropin (PMSG; Sigma-Aldrich, St. Louis, MO, USA) and then sacrificed 46 hours later. Cumulus-enclosed oocyte complexes (COCs) were recovered from ovaries by puncturing the preovulatory follicles with needles. Lactate-free M2 containing 0.2 mM 3-isobutyl-1-methyl-xanthine (IBMX; Sigma-Aldrich) was used to inhibit GVBD. Cumulus cells (CCs) were removed from COCs mechanically by aspiration with a fine-bore pipette. Mural granulosa cells (GCs) were recovered from preovulatory follicles.

To obtain MII oocytes, female mice were treated with 5 IU PMSG, followed by 5 IU human chorionic gonadotropin (hCG) after 46 hours. Superovulated MII oocytes were obtained from the oviduct 16 hours after hCG injection. CCs surrounding MII oocytes were removed by treating COCs with hyaluronidase (300 U/ml, Sigma-Aldrich).

### Preparation of *Txnip* dsRNA and microinjection

*Txnip*-A primers were used to amplify a region of *Txnip* cDNA, which then was cloned into pGEM-T Easy vector (Promega, Madison, WI, USA) and linearized with SpeI. RNA was synthesized using the MEGAscript RNAi Kit (Ambion, Austin, TX, USA) and T7 RNA polymerase. Single-stranded sense and anti-sense transcripts were mixed and incubated at 75°C for 5 minutes then cooled to room temperature. To remove contaminated single-stranded cRNA and DNA in the dsRNA samples, the preparation was treated with RNase (Ambion) and Dnase (Ambion), respectively, for 1 hour at 37°C. Formation of dsRNA was confirmed by 1% agarose gel electrophoresis. For microinjection, RNAs were diluted to a final concentration of 3.5 µg/µl.

Approximately 10 pl of dsRNA was microinjected into each GV oocyte cytoplasm in lactate-free M2 medium containing 0.2 Mm IBMX using a constant-flow system (Femtojet; Eppendorf, Hamburg, Germany). Buffer-injected oocytes were used as a sham control to assess injection damage.

### *In vitro* maturation of oocytes

Microinjected GV oocytes were cultured in lactate-free M16 medium containing 0.2 mM IBMX for 8 hours for degradation of target transcripts followed by culture in M16 medium for 16 hours in 5% CO_2_ at 37°C to determine the rate of maturation *in vitro*. Oocytes without GVs or polar bodies were scored as MI. Emission of the first polar body was used as an indicator of progression to MII. When high levels of lactate were used to treat control oocytes, five times of lactate was added to M16 medium (Sigma-Aldrich).

### Time lapse video microscopy

Time lapse video microscopy was performed to track phenotypic changes and the speed of oocyte maturation during *in vitro* culture. A time-lapse microscope (JuLI™; Digital Bio, Seoul, Korea) was placed in the incubator in 5% CO_2_ and 37°C and a culture dish containing oocytes was placed on the microscope stage. Images were automatically captured every 5 minutes for 16 hours and then sequential time lapse images were converted to movie files using JuLI operation software.

### Droplet culture for lactate production assay

To evaluate subtle changes in lactate concentration, we used lactate-free medium for culture. For droplet culture, 250 *Txnip* RNAi-treated oocytes and 250 control oocytes were placed in 20 µl droplets of lactate-free M16 and incubated for 16 hours under mineral oil in 5% CO_2_ at 37°C. Droplets and oocytes were removed and oocytes were mixed with lactate assay buffer. The culture medium and oocyte lysates were stored at −80°C until analysis. Changes in lactate concentration after *Txnip* RNAi treatment was measured by using a lactate colorimetric assay according to the manufacturer's instructions (Eton Bioscience, San Diego, CA, USA). Lactate concentrations were calculated from the standard curve.

### Evaluation of glucose uptake

A fluorescent glucose analogue, 2-(N-(7-nitrobenz-2-oxa-1,3-diazol-4-yl)amino)-2-deoxyglucose (2-NBDG; Molecular Probes, Eugene, OR, USA), was used to monitor glucose uptake during oocyte maturation. Oocytes from each group were incubated in M16 medium containing 200 µM NBDG for 20 minutes in 5% CO_2_ and 37°C. After thorough washing with M16 medium, oocytes were imaged at 488 nm by time lapse video microscopy according to the methods described in method of time lapse microscopy.

### Messenger RNA isolation, RT-PCR analysis and quantitative real-time RT-PCR

Messenger RNA was isolated from oocytes using the Dynabeads mRNA DIRECT kit (Dynal Asa, Oslo, Norway), according to the manufacturer's instructions. Briefly, oocytes were resuspended in 300 µl lysis/binding buffer and mixed with 20 µl prewashed Dynabeads oligo dT_25_ for annealing for 5 minutes at room temperature. After the beads were separated with a Dynal MPC-S magnetic particle concentrator and washed with buffer A twice followed by buffer B, poly(A)^+^ RNAs were eluted by incubation in 13 µl 10 mM Tris-HCl (pH 7.5) at 73°C for 2 minutes.

Purified mRNA and a 0.5 µg oligo (dT) primer were mixed and incubated at 70°C for 10 minutes, and cDNA was synthesized with Reverse transcriptase and RNase inhibitor (Promega). Single oocyte-equivalent cDNA was used as the template for PCR analysis. Primer sequences for the genes used in this study and its PCR conditions are listed in Table 1. The mouse *Txnip* gene has two variants, so we selected conserved sequences of these variants for primer design. PCR products were electrophoresed on a 1.5% agarose gel followed by analysis using an image analyzer (Gel Logic 112; Kodak, Rochester, NY, USA). For the comparison of the level of *Txnip* mRNA expression with that of the other genes, quantitative real-time RT-PCR was performed as previously described [19].

**Table 1:** Sequences of primers used in this study and RT-PCR conditions.

Genes	Accession no.	Primer sequences^a^	Annealing temperature	Product size
*Txnip-a*	NM_001009935.2	F : CAGCCAACTCAAGAGGCAAA R : ATTGGCAGCAGGTCTGGTCT	60°C	506 bp
*Txnip-b*	NM_001009935.2	F : TGGCTCCAAGAAAGTCATCC R : TTGAGAGTCGTCCACATCGT	60°C	229 bp
*Sebox*	NM_008759	F : AAAGCCAGGAGCCCTAAACT R : TTAGAAGTGGTCTACATTGG	60°C	334 bp
*Bcl2l10*	AF067660	F : CTCTGTGACTAGGCAGATCC R : GTCTCTAGGCTGGAGGACTT	60°C	551 bp
*Obox4*	AF461109	F : CCCTCATTGATCAACCCTTGG R : AGTTTTGGGTCATACTTGGAG	60°C	240 bp
*Glut1*	NM_011400.3	F : AAAGAAGAGGGTCGGCAGAT R : ACAGCGACACCACAGTGAAG	60°C	244 bp
*Glut2*	NM_031197.2	F : GCCTGTGTATGCAACCATTG R : GAAGATGGCAGTCATGCTC′	60°C	205 bp
*Glut3*	NM_011401.4	F : GAACCGATCTATGCCACGAT R : GCCAGGTCCAATCTCAAAGA	60°C	240 bp
*Glut4*	NM_009204.2	F : CAACAGCTCTCAGGCATCAA R : CTCAAAGAAGGCCACAAAGC	60°C	297 bp
*Glut5*	NM_019741.3	F : TCTCCGTTGGCAACTCATCT R : CCCCAAAGCTCTACCACAAA	60°C	201 bp
*Glut6*	NM_001177627.1	F : GAGGTCCATTGGGAGTTTGA R : AACTATCGCTGCATCCTGCT	60°C	228 bp
*Glut7*	NM_001085529.1	F : GATTCTCCTGCTGTCTGGCTAT R : GATGGATGGAAACGTCAACC	60°C	274 bp
*Glut8*	NM_019488.4	F : TTTCCCTCATGGTCTTCCAG R : AGGCTCTGGGTCAGTTTGAA	60°C	256 bp
*Glut9*	NM_001012363	F : GAAGTCCACATTGCTGGTCA R : TGCAGATGAAGATGGCAGTC	60°C	221 bp
*Glut10*	NM_130451.2	F : GGTTGGCTTTGGACCAGTAA R : TAGATGAAAGCCAGGCCAA	60°C	210 bp
*Glut12*	NM_178934.4	F : ACAGCAAGGGCTCGTTTATG R : TGACACCCCAGTTCATGCTA	60°C	294 bp
*Glut13*	NM_001033633.3	F : GATCAACGGTTCAGCTGTCA R : CTGAGCACGCATTTCCTGTA	60°C	278 bp
*Fshr*	NM_013523	F : TCCTTCATGGGACTGAGCTT R : AGAGGCTCCCTGCAAAACAT	60°C	165 bp
*Gdf9*	NM_008110	F : GGTTCTATCTGATAGGCGAGG R : GGGGCTGAAGGAGGGAGG	60°C	446 bp
*Gapdh*	BC092294	F : ACCACAGTCCATGCCATCAC R : TCCACCACCCTGTTGCTGTA	60°C	451 bp
*H1foo*	NM_008872	F : GCGAAACCGAAAGAGGTCAGAA R : TGGAGGAGGTCTTGGGAAGTAA	60°C	378 bp

a F, forward; R, reverse.

*Txnip-a* primers were used for preparation of dsRNA.

*Txnip-b* primers were used for confirmation target mRNA knockdown after RNAi treatment.

### Western blot analysis

Protein extract (50 oocytes per lane) was separated using 12% SDS-PAGE and transferred onto a polyvinylidene difluoride membrane (Bio-Rad Laboratories, Hercules, CA, USA). The membrane was blocked for 1 hour in Tris-buffered saline-Tween (TBS-T) containing 5% skim milk and then incubated with mouse monoclonal anti-TXNIP antibody (1∶400; MBL, Nagoya, Japan) or rabbit monoclonal anti-α-Tubulin antibody (1∶1000; Cell Signaling Technology, Danvers, MA, USA) in TBS-T. After incubation, membranes were incubated with horseradish peroxidase-conjugated anti-mouse IgG or anti-rabbit IgG (1∶1000; Sigma-Aldrich) in TBS-T for 1 hour at room temperature. After each step, the membranes were washed three times with TBS-T, and bound antibody was detected using an enhanced chemiluminescence (ECL) detection system (Amersham Biosciences, Piscataway, NJ, USA) according to the manufacturer's instructions. Protein levels were quantified by measuring the density of area for each band using Image J software (NIH). These values were then normalized to that of α-Tubulin and were expressed as a percentage of control oocytes.

### Immunofluorescence staining

Oocytes were fixed in PFA solution (4% paraformaldehyde and 0.2% Triton X-100) for 40 minutes at room temperature, washed three times in PVA-PBS for 10 minutes, and then stored overnight in 1% BSA-supplemented PVA-PBS (BSA-PVA-PBS) at 4°C. The oocytes were blocked with 3% BSA-PVA-PBS for 1 hour and incubated in 1% BSA-PVA-PBS containing mouse monoclonal α-Tubulin antibody (1∶100; Santa Cruz Biotechnology, Santa Cruz, CA, USA) at 4°C overnight. After washing three times in PVA-PBS, oocytes were incubated for 1 hour at room temperature with a second antibody diluted 1∶100 (fluorescein isothiocyanate-conjugated counterstained anti-mouse IgG; Sigma-Aldrich) in 1% BSA-PVA-PBS. After washing three times, they were incubated in a propidium iodide (PI) solution (1 mg/ml; Sigma-Aldrich) for 30 minutes to counterstain DNA, and then mounted between a microscope slide and a clean coverslip.

### Statistical analysis

Each experiment was repeated at least three times. Data were presented as mean ± SEM derived from at least three separate and independent experiments and were evaluated using one-way analysis of variance (ANOVA) and a log linear model. A value of p<0.05 was considered as statistically significant.

## Results

### Expression of *Txnip* during oocyte maturation

**Figure 1:**
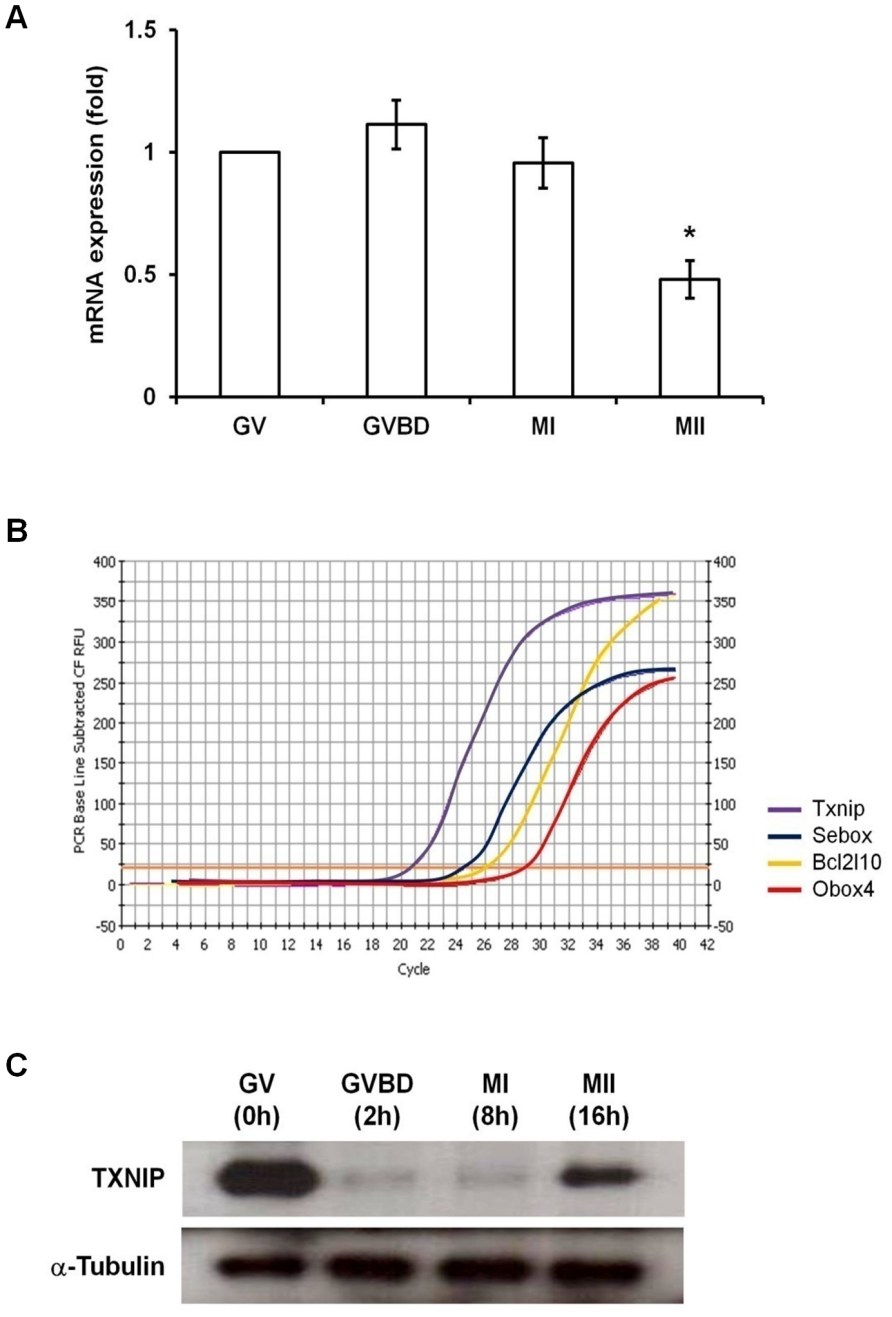
Expression of *Txnip* during oocyte maturation. (**A**) Expression of *Txnip* mRNA during oocyte maturation. For quantitative real-time RT-PCR, cDNA equivalent to a single oocyte was used as a template for amplification. GV, GVBD, MI, and MII oocytes were obtained after *in vitro* culture for 0, 2, 8, and 16 hours, respectively. Expression levels were calculated from C_T_ values after normalization with *H1foo* mRNA. Experiments were repeated at least three times, and data are expressed as mean ± SEM. Asterisk represents statistically significant differences compared with GV oocytes (*p*<0.05). (**B**) Comparison of C_T_ value of *Txnip* with that of other genes studied in our laboratory (*Sebox*, *Bcl2l10*, and *Obox4*). (**C**) Expression of TXNIP protein during oocyte maturation. Proteins were extracted from 50 mouse oocytes at each stage. α-Tubulin was used as a loading control.

During oocyte maturation process, *Txnip* mRNA expression was higher in GV, GVBD, and MI than in MII oocytes (Fig. 1A). Likewise, a higher level of endogenous TXNIP protein expression was detected at the GV stage compared to the MII stage (Fig. 1B). Notably, cycle threshold (C_T_) value from quantitative real-time RT-PCR ranged from 21 to 23 indicating that *Txnip* transcripts are extremely abundant in GV oocytes compared to other genes previously studied in our laboratory such as *Sebox* [19], *Bcl2l10* [20], and *Obox4* [21], which had C_T_ values of 24, 26, and 29, respectively (Fig. 1B). Interestingly, TXNIP protein expression was barely present at the GVBD and MI stages although *Txnip* mRNA was still highly expressed in both stages, at a level similar to that of GV oocytes, (Fig. 1C). Reasons of the discrepancy between the expression amount of mRNA and protein (Fig. 1A and 1C) are inexplicable at this moment.

### Sequence-specific degradation of *Txnip* and resulted outcomes

#### Meiotic arrest at MI

**Figure 2:**
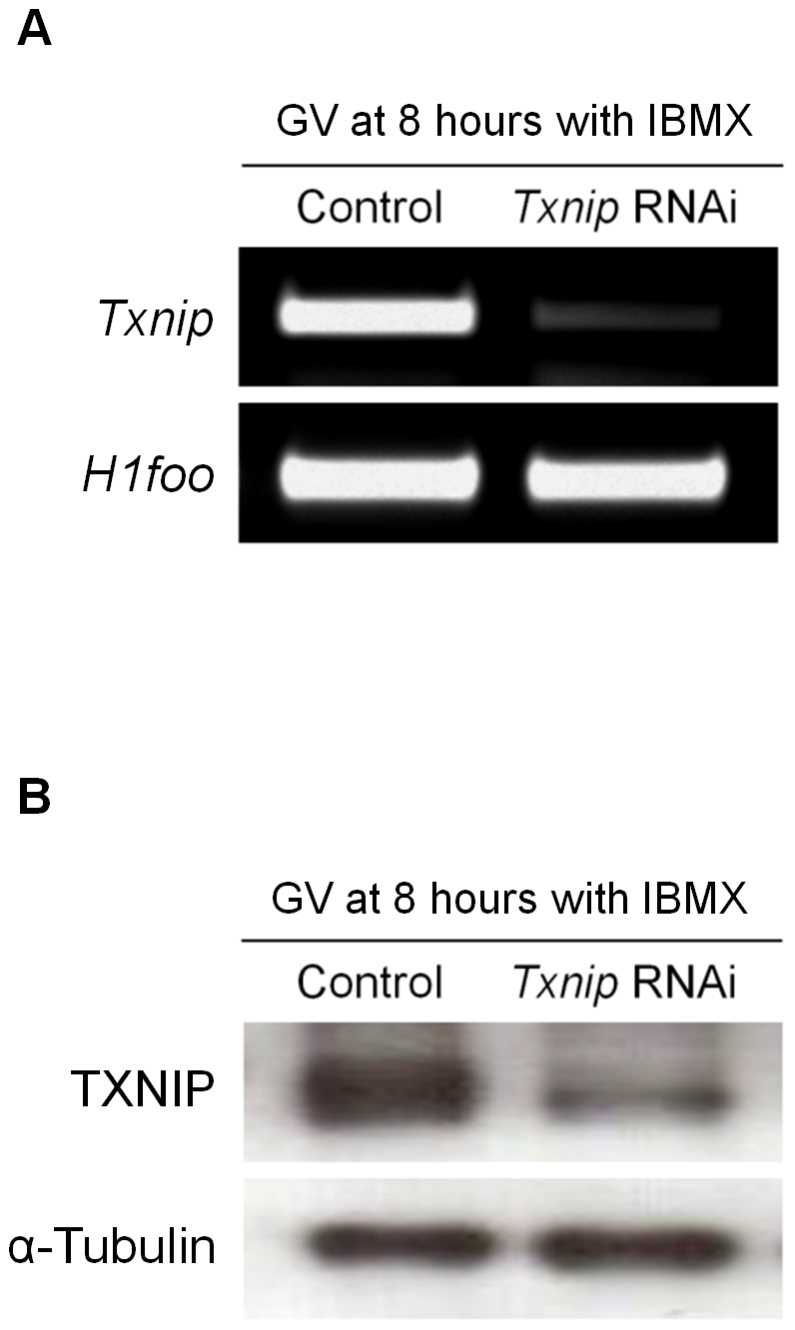
*Txnip* RNAi-mediated degradation of *Txnip*. Specific depletion of (**A**) *Txnip* mRNA and (**B**) TXNIP protein after RNAi. *H1foo* was used as an internal control for oocytes, while α-Tubulin was used as a loading control. Protein lysates of 50 oocytes were loaded per lane for Western blotting. Experiments were repeated at least three times.

For sufficient degradation of *Txnip* mRNA, oocytes were incubated in IBMX-supplemented M16 medium before *in vitro* maturation in M16 alone. As a result, complete *Txnip* mRNA degradation was verified by 8 hours after microinjection of *Txnip* dsRNA (Fig. 2A). To confirm depletion of TXNIP protein expression, Western blot analysis was performed, and results showed that TXNIP protein level decreased markedly with *Txnip* RNAi treatment (Fig. 2B). The residual TXNIP protein found in Fig. 2B is thought as an endogenous protein translated before *Txnip* RNAi was performed. These results suggest that *Txnip* RNAi caused sequence-specific suppression of *Txnip* expression. The maturation rate of *Txnip* RNAi-treated GV oocytes to MII stage (5.3%) significantly decreased compared with that of oocytes in control (81.25%) or buffer-injected (76.3%) groups. Most of the oocytes were arrested at the MI stage (79.5%) after *Txnip* RNAi (Table 2).

**Table 2:** *Txnip* RNAi-treated oocytes arrested at the MI stage during *in vitro* maturation.

	Number of oocytes (%)			
Treatment	Total	Germinal vesicle (GV)	Metaphase I (MI)	Metaphase II (MII)
Control	96	0 (0)	18 (18.75)	78 (81.25)
Buffer-injected	97	0 (0)	23 (23.7)	74 (76.3)
*Txnip* RNAi	132	20 (15.2)	105 (79.5)^a^	7 (5.3)^a^

a Values with statistical significance (*p*<0.05) compared to control or buffer-injected groups.

#### Granule formation with retarded cytoplasmic streaming during *in vitro* maturation

**Figure 3:**
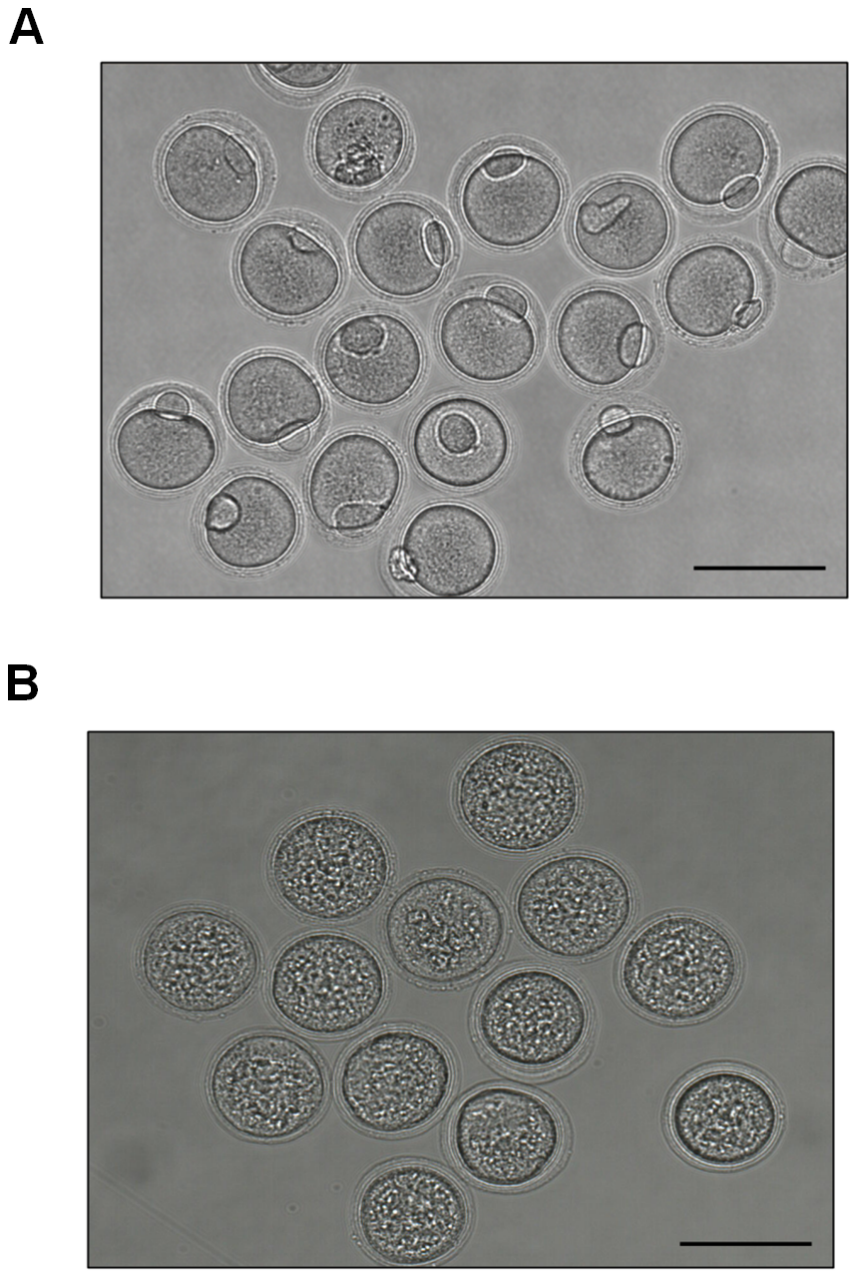
*Txnip* RNAi treatment resulted in MI arrest and granule formation. Microphotographs of (**A**) control oocytes and (**B**) *Txnip* RNAi-treated oocytes after *in vitro* culture for 16 hours in M16 medium following 8 hours incubation in IBMX-supplemented M16 medium. Bars = 100 µm.

While control oocytes released the first polar body to complete meiosis within 16 hours (Fig. 3A), *Txnip* RNAi-treated oocytes were arrested at the MI stage (Fig. 3B). During *in vitro* maturation after *Txnip* RNAi treatment, enormous amount of intracellular granules were formed in the cytoplasm of oocytes (Fig. 3B). In addition, we observed that the cytoplasmic streaming was retarded in *Txnip* RNAi-treated oocytes compared to the fast cytoplasmic streaming found in the control oocytes (Video S1 and Video S2; Control oocytes and *Txnip* RNAi-treated oocyte, respectively).

#### Affected spindle and chromosomal organization

**Figure 4:**
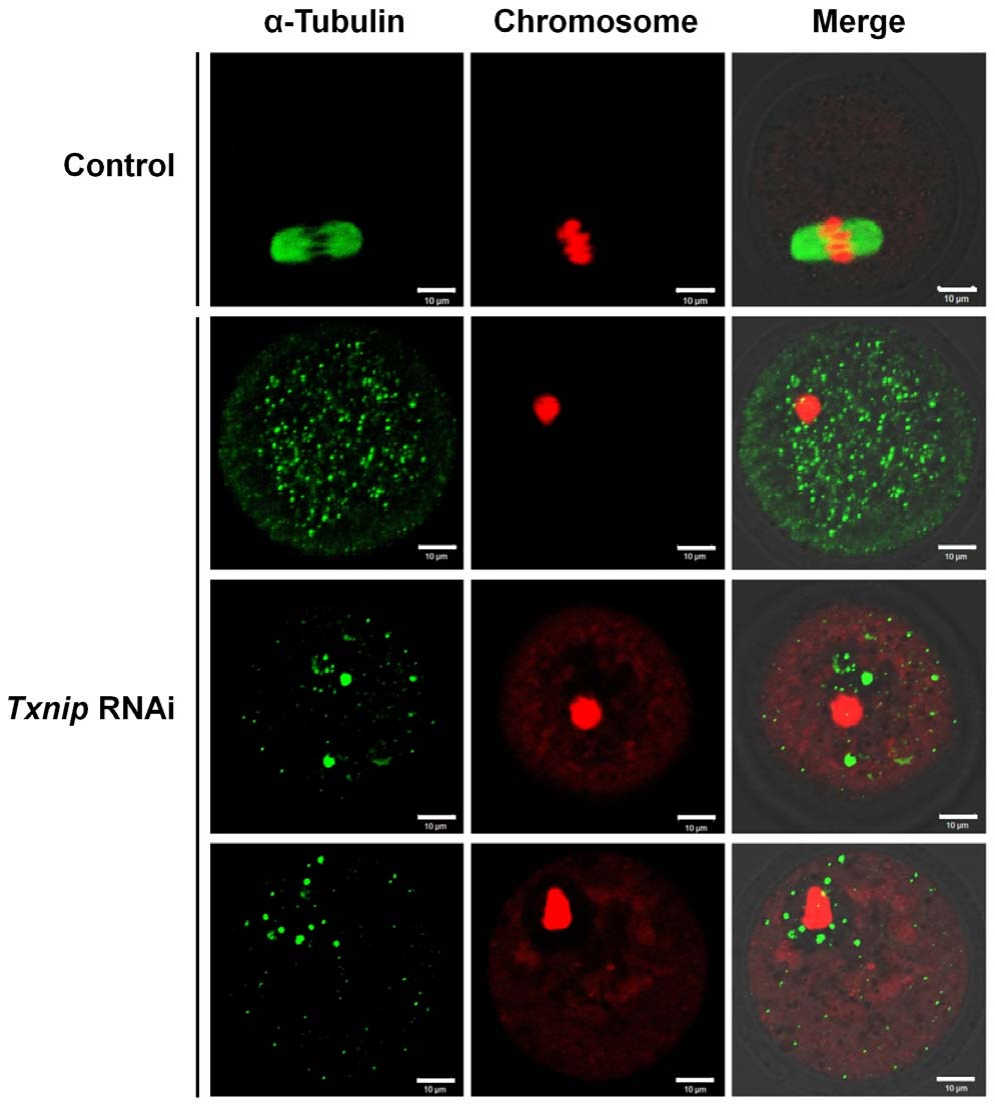
Immunofluorescence staining of spindles and chromosomes. Spindles were stained with α-Tubulin antibody (green) and chromosomes were counterstained with propidium iodide (red). Control MI oocytes were cultured for 8 hours *in vitro*. MI-arrested oocytes of *Txnip* RNAi group showed aggregated chromosomes at the center of the oocytes and scattered dots of spindle. Bars = 10 µm.

While control MI oocytes showed normal, barrel-shaped spindles and well-aligned chromosomes at the metaphase plate during oocyte maturation, the *Txnip* RNAi-treated oocytes exhibited condensed chromosomes in one mass with scattered tubulin dots throughout the cytoplasm (Fig. 4).

#### Increased lactate production with glucose uptake

**Figure 5:**
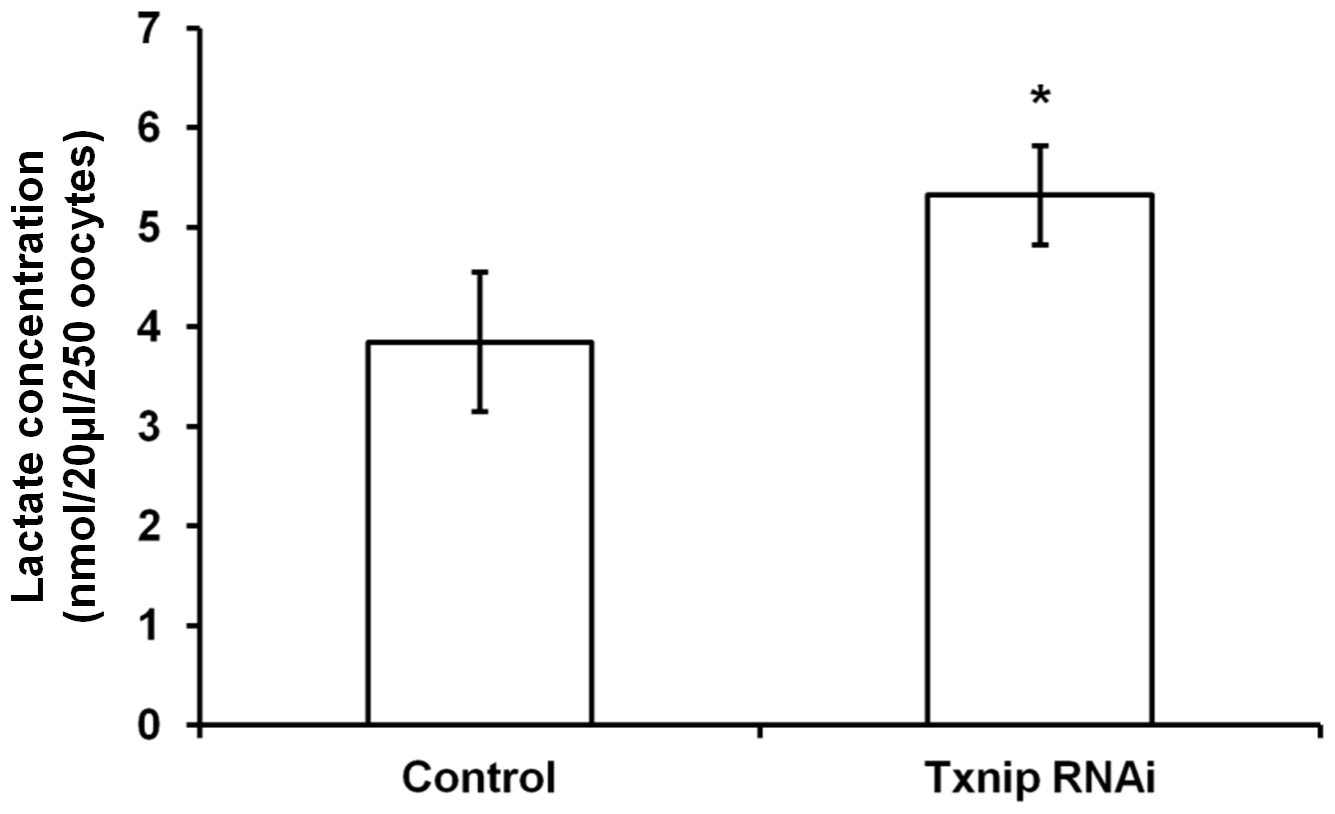
Lactate production was increased in *Txnip* RNAi-treatment oocytes. Lactate production was measured by a lactate colorimetric assay kit after droplet culture in 20 µl of M16 medium. The y-axis indicates the concentration of lactate from a total of 250 oocytes used for each group. Asterisk indicates statistically significant difference compared to the control (p<0.05).

**Figure 6:**
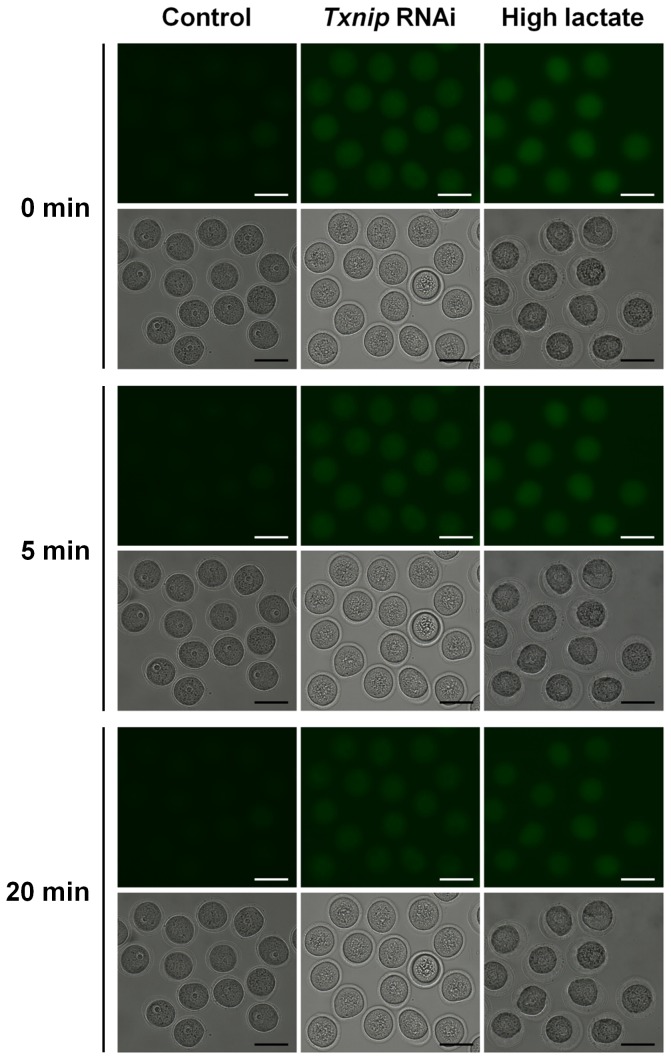
Results of 2-NBDG treatment to visualize glucose uptake into oocytes. DOs were incubated in M16 medium containing 200 µM 2-NBDG for 20 minutes followed by *in vitro* maturation in M16 medium for 16 hours, and oocytes were imaged for fluorescence quantification using time lapse microcopy. Fluorescence intensities of 2-NBDG in *Txnip* RNAi-treated oocytes and high concentration lactate-treated oocytes were stronger than control oocytes. Bars = 100 µm.

Because *Txnip* was known as a negative regulator of lactate production [14], we measured the lactate production during *in vitro* maturation. As we expected, the concentration of lactate was significantly increased by 39.5% in *Txnip* RNAi-treated oocytes (Fig. 5). Because of increased lactate production by *Txnip* RNAi treatment, we presumed that glucose uptake into oocytes were also upregulated by *Txnip* RNAi. Following sufficient time (8 hours) for *Txnip* knockdown in IBMX-supplemented M16 medium, we directly visualized glucose uptake using a non-metabolizable fluorescent glucose analogue, 2-NBDG [22], [23]. Within the first 5 minutes rapid NBDG uptake occurred and accumulated maximally after 15–20 minutes. As soon as oocytes were transferred to the M16 medium, *Txnip* RNAi-treated oocytes showed twice as strong fluorescence intensity compared with control oocytes as depicted in Figure 6. These results suggested that glucose uptake into oocytes increased during degradation of *Txnip* mRNA in IBMX-supplemented medium.

**Figure 7:**
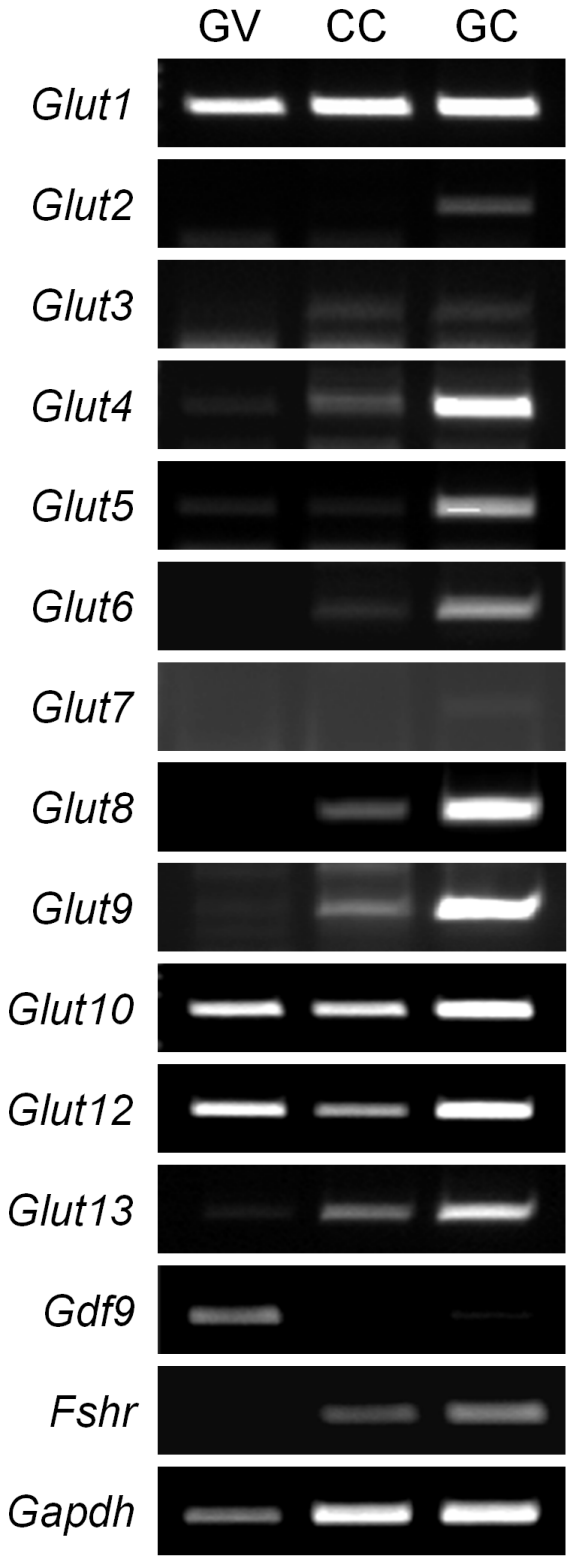
Expression of *Glut* family members in mouse oocytes and follicular cells. PCR was carried out by using cDNAs equivalent to a single-oocyte, CCs obtained from one oocyte and GCs obtained from one follicle. Sequences for *Glut* 11 and 14 have not been found in mice to date. GV, denuded GV oocytes; CC, cumulus cells; GC, granulosa cells. *Gdf9* and *Fshr* were used as markers for oocytes and granulosa cells, respectively. *Gapdh* was used as an internal control. Experiments were repeated at least three times.

It has long been reported and accepted that the oocytes are deficient in their ability to utilize the glucose thus require the surrounding CCs and GCs to utilize the glucose [22], [24]. According to the contradiction between this concept and our results in Figure 6, we decided to evaluate the existence of glucose transporters (*Gluts*) in the follicular cells including oocyte itself. As depicted in Figure 7, CCs and GCs express most of Glut family members. Expression of *Glut* 2 and 7 was not or weakly detected in CCs and GCs, respectively. In addition, we observed that oocytes also express substantial level of *Glut* 1, 10, and 12. Therefore, we concluded that glucose uptake can occur in oocytes that may be not actively used in nature, and the increased glucose and lactate in the oocyte cytoplasm after *Txnip* RNAi may induce the gluconeogenic pathway in oocytes and those intracellular granules may indicative of that.

### Effect of high lactate treatment was comparable to *Txnip* RNAi treatment

We investigated how the high concentration of lactate affects oocyte maturation. The addition of five folds higher lactate to the culture medium resulted in a remarkable reduction of cytoplasmic streaming compared to the control oocytes with increased formation of small granules in the oocyte cytoplasm (Video S3, Fig. 6, column of High lactate). As we presumed, the glucose uptake increased and that was similar to the phenomena found in *Txnip* RNAi-treated oocytes.

## Discussion

In the present study, we firstly report the expression and function of *Txnip* in mouse oocytes. *Txnip* RNAi treatment increased oocytes' glucose uptake and lactate production and resulted in retarded cytoplasmic streaming with intracellular granule formation, abnormal spindle assembly, and chromosome arrangement.

Both abundant mRNAs and proteins are accumulated in the oocytes during oocyte development. Wang and colleagues reported a number of differentially expressed proteins among the different stages of mouse oocytes [25]. They demonstrated that the abundant proteins in GV oocytes are mainly related to actin binding, the microfilament motor, primary transporters, and amino acid transporters. In addition, many metabolism-related proteins were expressed in GV oocytes for supporting oocyte maturation [25]. We found that *Txnip* was highly expressed in GV oocytes than in MII oocytes in this study. Thus, we focused the investigation to the role of *Txnip* in regulation of glucose metabolism during oocyte maturation. *Txnip* expression has been reported in bovine CCs [26]. In that report, *Txnip* expression was lesser in the case of *in vitro* than *in vivo* maturation and the authors suggested that may result in decreased supply of glucose for oocytes during *in vitro* maturation [26].

Most interestingly, one of the notable phenotypic features of *Txnip* RNAi was the formation of intracellular granules in the oocyte cytoplasm. Accordingly, our first question was what these granules were and why they were formed in response to *Txnip* RNAi treatment. Mammalian oocytes and embryos contain glycogen granules as stored forms of glycogen, and/or lipid droplets, which are an energy source for the growth and development of the embryos, and the amount of such inclusions in an embryo varies with the stage of development [27]. Glucose is incorporated into glycogen, the end product of glucose metabolism [28]. Prior to glycogen synthesis, glucose is converted into lactate and lactate is synthesized into glycogen molecules via the gluconeogenic pathway [29]. *Txnip* has been reported to inhibit glucose uptake and lactate production [14]. Based on these previous reports, we assumed that increased glucose uptake and lactate production by *Txnip* RNAi would lead to the formation of glycogen granules in oocytes. To prove this hypothesis, we measured glucose uptake and lactate production after *Txnip* RNAi, and as we anticipated, *Txnip* RNAi-treated oocytes showed increased glucose uptake and lactate production compared with control oocytes. Thus, we concluded that the *Txnip* RNAi treatment resulted in enhanced lactate production by increased glucose uptake and this state promoted the formation of intracellular granules.

We used denuded oocytes when we measure glucose uptake and lactate production, since we used them for microinjection of dsRNA. By using 2-NBDG, we identified glucose uptake in denuded oocytes suggesting that glucose can enter oocytes in the absence of CCs. In fact, we verified the expression of *Glut* family members in denuded oocytes as well as in follicular cells suggesting the oocytes' ability of glucose uptake. It has long been known that oocytes are deficient in their ability to utilize glucose and require CCs to take up glucose via *Gluts* system and metabolize it into pyruvate which then is provided to oocytes [22], [24]. According to our results, we verified that the oocytes has the machinery for glycolytic metabolism and can uptake glucose. The carrier-mediated uptake of pyruvate and glucose in human and mouse oocytes has been reported [30].

Our second question was why cytoplasmic streaming was retarded after *Txnip* RNAi treatment. Cytoplasmic streaming, also known as cyclosis, is the movement of cytoplasm which transports cytoplasmic nutrients, microscopic particles, and organelles toward different parts of the cell where they are needed [31]. Cytoplasmic streaming is driven by actin filaments in plant cells [32]. In C. elegans oogenesis, cytoplasmic streaming driven by the actomyosin cytoskeleton has been shown to play a role in oocyte enlargement and both microfilaments and microtubules migrate in the direction of actin movement [33], [34]. Moreover, recent reports have suggested that actin movements induce cytoplasmic streaming in mouse oocytes [35], [36].

**Figure 8:**
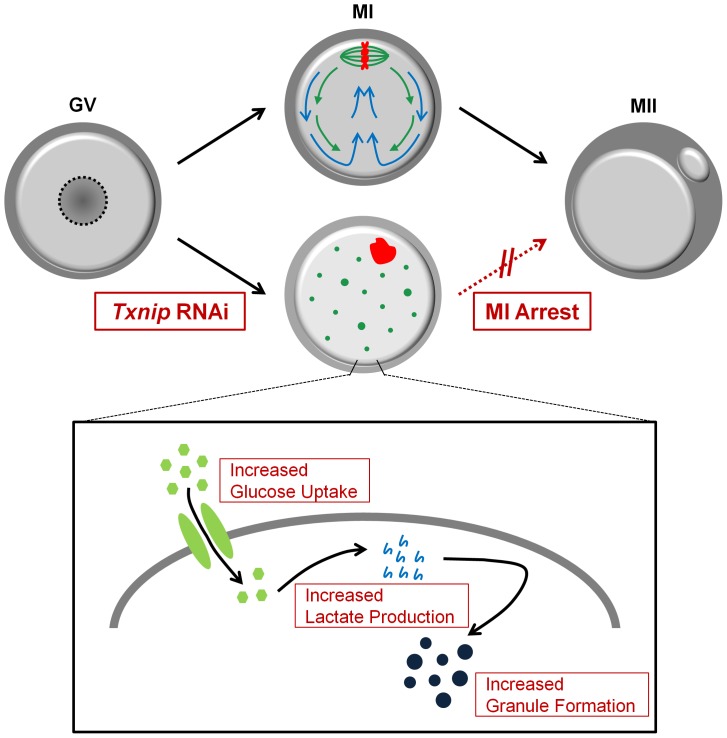
*Txnip* RNAi affects meiotic cell cycle by changing glucose metabolism and cytoplasmic streaming in mouse oocyte. In control oocytes, actin flow (green arrow) leads to dynamic cytoplasmic streaming (blue arrow) and both of them cause spindle migration in MI stage necessary to the first polar body extrusion in completing the meiosis I [41]. In the case of *Txnip* RNAi-treated oocytes, however, glucose uptake and lactate production was increased and as a result, intracellular granule formation was increased. This increased granule formation disturbed actin flow and cytoplasmic streaming, and led to abnormal spindle assembly (green dots), aggregated chromosomes (red figure), and finally, MI arrest of the meiotic cell cycle of the oocytes. GV: germinal vesicle; MI: metaphase I; MII: metaphase II oocytes.

Interactions between microtubules and actin filaments are notably important in oogenesis [37]. Cytoplasmic actins play a role in nuclear anchoring [38], meiotic spindle positioning that is required for asymmetric cell division [39], and chromosome assembly [40]. In the present study, although we did not directly observe actin movement, scattered dots of tubulin and aggregated chromosomes support the idea that actin flow was disturbed by *Txnip* RNAi treatment. Therefore, we concluded that the *Txnip* RNAi contributes to abnormal actin flow and retarded cytoplasmic streaming. We supposed that granules formed by *Txnip* RNAi treatment physically obstructed normal microtubule-actin filament movement. The results are summarized in Figure 8.

In conclusion, our findings demonstrated that *Txnip* is a critical regulator of glucose metabolism and regulates cytoplasmic streaming and meiotic maturation of the mouse oocytes. It is worth to note that our results provide the first evidence of *Txnip* expression and its role in mouse oocytes. Further study of molecular mechanisms involved in the function of *Txnip* would contribute towards elucidating the association between metabolic abnormalities and female fertility.

## Supporting Information

Video S1 **Time-lapse microscopy of control oocytes during in vitro maturation.**
(AVI)

Video S2 **Time-lapse microscopy of** ***Txnip*** **RNAi-treated oocytes during** ***in vitro*** **maturation.** The cytoplasmic streaming was retarded in *Txnip* RNAi-treated oocytes compared to the relatively fast cytoplasmic streaming found in the control oocytes (Video S1).
(AVI)

Video S3 **Time-lapse microscopy of high lactate-treated oocytes during** ***in vitro*** **maturation.** The treatment of higher lactate to the culture medium resulted in a retarded cytoplasmic streaming compared to the control oocytes (Video S1) with increased formation of small granules in the oocyte cytoplasm. This result was similar to the phenomenon found in *Txnip* RNAi-treated oocytes (Video S2).
(AVI)
